# Annotations

**Published:** 1891-06-13

**Authors:** 


### Annotations.

A few weeks ago we discussed the "eight hours a day" question from the standpoint of physiology. The conviction was then expressed that all out-door workers, and nearly all indoor, could work ten hours a day without any disadvantage, under normally healthy conditions of atmosphere and general surroundings. The exactly anthithetic question of sixteen working hours a day is now forced upon public attention by the strike of the London 'busmen. Into the general merits of the dispute between the men and their employers we do not enter; but a very little knowledge of physiology is sufficient to teach any man that a working day of sixteen hours is entirely fatal to the worker. The nervous system, the digestive system, and the muscular system are alike put to undue strain, and cannot fail to undergo degeneration. It is a physiological impossibility for any man to work sixteen hours a day without a breakdown; and the longer he attempts such unnatural work the more complete and final will be the breakdown when it comes. A working day of twelve hours is the maximum which any class of workers should be called upon to agree with. The general principle ought to be universally recognised that reasonable work is such a measure of exertion as can be performed, not only without mental and physical injury, but with mental and physical advantage. Work that breaks down and kills the worker should not be undertaken at all without the sternest compulsion of necessity. All those stupid old maxims which affirm that "work kills nobody," that "it is better to wear out than to rust out," that "he who is quick at meat is quick at work," that "the men and boys of the present generation do not work half so hard as their fathers and grandfathers did"—all these are mere silly nonsense and twaddle. Whilst we have the greatest possible respect for experience and its teachings, we insist upon the necessity of such an experience as will stand the test of reason and science. Reason and science make it as plain as the sun at noon, that twelve hours a day are the maximum for hard work; and that in all cases where it is possible, one or two hours less than the twelve are a distinct advantage to the worker.

Sixteen Hours a Day.

To mention such an institution as a home for the dying, for a vast population like that of London, is sufficient to convince every experienced person of the need for its existence. It is probable that most people think there is at least one such home in the metropolis, and that there may possibly be several. We are so accustomed to the idea that every human necessity is provided for in our Imperial Capital that we can hardly bring ourselves to believe that so urgent and painful a need as a shelter for those who are dying and destitute, with kind ministrations for the last few days or weeks of weary life, is practically not to be found. But such, nevertheless, is the fact. Notwithstanding the large number and great variety of charitable institutions which our benevolent fellow-countrymen have founded, they have strangely overlooked the necessity of a home for the dying. It will give great satisfaction to many readers to be informed that an effort has at last been commenced to provide a suitable asylum for those who, though having but a few months to live, are homeless and without resource. The new movement is commenced under very favourable auspices, and has associated with it such well known philanthropists as Sir Robert Phayre, Mr. J. Tritton, Mr. T. A. Denny, Mr. Pearce Gould, F.R.C.S., Dr. Habershon, F.R.C.P., Mr. Hugh Matheson, Colonel J. F. Morton, the Reverend H. W. Webb-Peploe, the Rev. Dr. Sinclair Paterson, Mr. Nevile Sherbrooke, and others. The Committee propose to raise at once the sum of ten thousand pounds. Nearly two thousand have already been guaranteed from private sources. So far back as five years ago a small "home" was started with ten beds; and this has been maintained largely by some members of the Committee and their friends. By way of proving how urgent is the need of a home for the dying it is stated that even at this little known private home no fewer than three hundred applications for admission have been made annually. Of those who have applied it has only been possible to admit about forty each year: the remaining two hundred and sixty have been sorrowfully refused. It is not necessary to make urgent pleading on behalf of an institution of this kind. When a man is dying the world owes him at least the opportunity of dying in peace, and with soothing ministrations for this world and the world that is to come. Let his life have been what it may, he is laying it down, in poverty, perhaps in pain, possibly in bitter disappointment and brokenness of heart. Give him a peaceful haven of rest for the sake of charity. It is needless to say that we commend this Home for the Dying to all who have means and a kindly heart. The address of the honorary Secretary is Friedenheim, 133, Mildmay Road, London, N.

A Home for the Dying.

If any remedy in the Pharmacopœia may ever be considered to have established a secure reputation, opium, one would think, could be named as one such remedy. The older practitioners of all classes, from the great London consultant down to the parish doctor in the remotest country village, often worked their miracles with opium. At the present day, too, the man of large bedside experience, whose purpose it is to cure or relieve his patient, and not merely to give him a sounding exposition of the "interesting" diagnostic features of his case, finds that opium is one of the most helpful and extensively beneficial of all the substances that are known to him in the whole region of therapeutics. But, according to Dr. Samuel Wilks, consulting physician to Guy's Hospital, and a man of practically unlimited experience, this most invaluable of all remedies has, to a large extent, gone out of use, in consequence of the changed medical fashions of the day. "When a student, between forty and fifty years ago," says Dr. Wilks, "I was taught that opium was a remedy of the most potent and valuable kind, having of necessity a widespread utility owing to its action on the nervous system, whereby it diminished the functional activity of every organ of the body, whether this was brain, liver, kidney, intestine, or uterus. It consequently lessened the wear and tear of the tissues, and so tended in many conditions to sustain or prolong life. In a similar manner it would arrest many morbid actions depending upon acute cell growth, as in inflammations, and was a remedy, therefore, in all diseases terminating in 'itis. This was illustrated by cases which could by confirmed by the eye, as the rapid subsidence of a sore throat under a dose of laudanum, or the quickly-healing large sloughing ulcer in the leg under the continued action of the same drug—a treatment, I may remark, largely pursued by Mr. Aston Key. What I was then taught I have never had any reason to dispute, for a long practice has confirmed me in the truth of these doctrines, and I have continued the use of opium and morphine as heretofore, having found no other drugs to replace them." An appeal to any really experienced physician of mature age would confirm these statements in almost every detail. The point of Dr. Wilks' contention, be it observed, is not only that opium is a valuable remedy, but that in certain specified cases no other remedy has yet been discovered which is equally valuable. But, notwithstanding this, opium is given up, and other less efficient remedies are substituted. Can this be called medical progress? Do the youthful and modern physicians who thus despise the teachings of experience deserve praise or blame at our hands? Are they scientists working out sound inductions, as Bacon was a scientist; or are they fashionable "faddists" following every whim of the moment as the weak-minded "society" woman follows the ridiculous vagaries of the fashion-plate? It is to be feared that the youthful physicians and surgeons are not the only offenders or the only enemies of rational medical progress. There are a certain number of their elders who are so mortally afraid of not being "up to date" that they would rather use a less efficient remedy if it happened to be new than one that was more efficient if it happened to be old. What a sorry and contemptible want of scientific backbone is this! Things new by all means let us have provided they are also true; but things true, whose truth and value have been proved by twice ten thousand experiences, must always be held fast unless we wish to show to the world that the medical intellect is of the kind possessed by children and fools.

Medical Fashions.

